# Transcriptome Analysis of *Litopenaeus vannamei* in Response to White Spot Syndrome Virus Infection

**DOI:** 10.1371/journal.pone.0073218

**Published:** 2013-08-26

**Authors:** Xiaohan Chen, Digang Zeng, Xiuli Chen, Daxiang Xie, Yongzhen Zhao, Chunling Yang, Yongmei Li, Ning Ma, Ming Li, Qiong Yang, Zhenping Liao, Hui Wang

**Affiliations:** Guangxi Key Laboratory of Aquatic Genetic Breeding and Healthy Aquaculture, Guangxi Institute of Fisheries, Nanning, China; Macquarie University, Australia

## Abstract

Pacific white shrimp (*Litopenaeus vannamei*) is the most extensively farmed crustacean species in the world. White spot syndrome virus (WSSV) is one of the major pathogens in the cultured shrimp. However, the molecular mechanisms of the host-virus interaction remain largely unknown. In this study, the impact of WSSV infection on host gene expression in the hepatopancreas of *L. vannamei* was investigated through the use of 454 pyrosequencing-based RNA-Seq of cDNA libraries developed from WSSV-challenged shrimp or normal controls. By comparing the two cDNA libraries, we show that 767 host genes are significantly up-regulated and 729 genes are significantly down-regulated by WSSV infection. KEGG analysis of the differentially expressed genes indicated that the distribution of gene pathways between the up- and down-regulated genes is quite different. Among the differentially expressed genes, several are found to be involved in various processes of animal defense against pathogens such as apoptosis, mitogen-activated protein kinase (MAPK) signaling, toll-like receptor (TLR) signaling, Wnt signaling and antigen processing and presentation pathways. The present study provides valuable information on differential expression of *L. vannamei* genes following WSSV infection and improves our current understanding of this host-virus interaction. In addition, the large number of transcripts obtained in this study provides a strong basis for future genomic research on shrimp.

## Introduction

Pacific white shrimp (*Litopenaeus vannamei*) has become the most extensively cultured crustacean species in the world due to its high-yield and low-demand for concentration of salt [1]. Over the last two decades, viral diseases have seriously threatened the shrimp aquaculture industry [2]–[5]. Among the viral diseases, white spot syndrome virus (WSSV) is the most important viral pathogens of shrimp farming, causing high mortality and large economic loss [6]. WSSV is an enveloped virus with a large, double stranded, circular genome (∼300 kb) [7], [8] and has a wide host range, including many aquatic crustaceans and even aquatic insects [9], [10]. In cultured shrimp, WSSV causes a cumulative mortality can reach 100% within 3–10 days [4]. To date, no effective cure for this disease has yet been found. As a result, the response of shrimp to viral pathogens has become an increasingly important subject for study [11]. An understanding of the molecular responses and defense mechanism in shrimp against pathogens will promote the development of effective techniques to prevent viral diseases of shrimp.

In recent years, some attempts have been made to investigate the effects of viral infection on shrimp transcriptome using the suppression subtractive hybridization (SSH) or the cDNA microarray technology [12]–[14]. Several immune-related proteins in shrimp, such as hemocyanin [15], [16], lectins [17], [18], antimicrobial peptide [19] and superoxide dismutase [20] have been identified. However, such studies have been hampered by a relative lack of molecular genetic information and tools suitable for high throughput assessment of gene expression [11].

Recently, several next-generation sequencing technologies, such as the 454 Life Sciences (Roche) pyrosequencing platform, the Illumina Genome Analyzer and the Applied Biosystems Solid platform, provide a rapid, cost-effective and high-throughput tool for sequencing of the transcriptome (RNA-Seq) [21], [22]. The RNA-Seq permits deep, robust assessments of transcript abundances and transcript structure [23]. When gene expression profiling is applied to the interaction between WSSV and shrimp, insights into the mechanisms this virus use to suppress and subvert the host defense response can be made.

In this study, we used 454 pyrosequencing to conduct a comparative transcriptome profiling analysis between the hepatopancreas of WSSV-infected *L. vannamei* shrimp and uninfected controls, with the aim of investigating candidate immune-related genes in shrimp, improving the current understanding of the host-virus interaction, and providing a substantial dataset that increases the publicly available DNA sequence resources for this crustacean species.

## Materials and Methods

### Experimental shrimp and WSSV challenge

The experiment was conducted using a specific pathogen-free (SPF) shrimp *L. vannamei* strain (National and Guangxi Shrimp Genetic Breeding Center, Guangxi Province, China). The shrimp (11–12 g body weight) were maintained in the environmentally controlled 1000-liter glass salt water tanks (32-ppt salinity, 25 to 26°C) and fed an artificial pellet feed. In the challenge experiment, a batch of shrimp was divided into a challenge group and a normal control group. The challenge group contained 40 shrimp, and the control group contained 20 shrimp. Shrimp in the challenge group were fed once a day for 3 consecutive days with minced WSSV-infected tail tissue at 5% of their body weight (a dose of about 1×10^5^ WSSV copies/g sufficient to cause 100% mortality in 5–7 days). The WSSV dose was determined in a previous experiment, in which RT-PCR method and reference samples containing gradient concentrations of WSSV were used. The WSSV strain was isolated by our laboratory in 2008 from a *L. vannamei* shrimp in China. In the challenge experiment, the mortality rate of challenge group was 0, 5% and 32.5% at 24 h, 48 h and 72 h, respectively, after WSSV challenge. In parallel, shrimp in the control group were fed once a day for 3 consecutive days with minced PCR-confirmed [24] healthy tail tissue at 5% of their body weight [25]. The hepatopancreas tissues of shrimp (20 shrimp each group) were taken at 72 h post challenge and stored in liquid nitrogen (−196°C) until RNA isolation.

### RNA extraction, cDNA library construction and RNA-seq

Total RNA was extracted using TRIzol reagent (Qiagen) following the manufacturer's instructions. RNA concentrations were measured using a spectrophotometer, and integrity was ensured through the analysis on a 1.5% (w/v) agarose gel. After RNA extraction, mRNAs were purified using the PolyATtract® mRNA Isolation Systems (Promega) and concentrated using the Reasy RNA Cleaning Kit (Qiagen). Equal amounts of the high-quality mRNA samples from each group were then pooled for cDNA synthesis and sequencing. The pooled mRNAs were fragmented into small pieces using RNA fragment reagent (Qiagen), and the pieces were collected using the Reasy RNA Cleaning Kit (Qiagen). Subsequently, cleaved RNA fragments were copied into first strand cDNA using MMLV reverse transcriptase and random primers. This was followed by second strand cDNA synthesis using DNA polymerase I and RNase H. Library construction and a 1/2 plate pyrosequencing run was performed by Beijing Autolab Biotechnology Co., Ltd.(China) on a 454 GS FLX system (Roche) according to the manufacturer's instructions.

### *De novo* assembly and gene annotation

Before the assembly, the raw sequencing reads were qualities trimmed and adaptor sequences removed using the SeqClean program (http://compbio.dfci.harvard.edu/tgi/software). The screened high-quality sequences (cleaned reads) were de novo assembled using the iAssembler program (http://bioinfo.bti.cornell.edu/tool/iAssembler) with default parameters [26]. The overall assembly was performed using the combined sequence data from both the WSSV-infected and the control samples. The contigs and singletons were generally referred to as unigenes. Open reading frames (ORF) of each unigene were identified by getorf (http://emboss.bioinformatics.nl/cgi-bin/emboss/getorf). For functional annotation analysis, all unigenes were compared against sequences in NCBI non-redundant (nr) protein and UniProtKB/Swiss-Prot (UniProt release 2013_07 - Jun 26, 2013) [27] database using the BLASTX programs (E-value <10^−5^) [28]. Genes were tentatively identified according to the best hits against known sequences. Functional annotation by gene ontology terms (GO) was analyzed using a BLAST2GO program (http://www.BLAST2go.org/). The COG and KEGG pathway annotations of unigenes was performed using the BLASTX software against the COG database and the KEGG database, respectively [29], [30].

### Identification of differentially expressed genes

For differential gene expression analyses, the transcript levels were measured as RPKM (Reads Per Kilobase of exon model per Million mapped reads) values to determine relative transcript abundance. Statistical comparison between two different libraries was conducted using a web tool IDEG6 (http://telethon.bio.unipd.it/bioinfo/IDEG6_form/) [31]. FDR (false discovery rate) <0.001 was used as the threshold of p-value in multiple test to judge the significance of gene expression difference [32]. Genes were considered differentially expressed in a given library when the p-value was less than 0.001, and a greater than two-fold change in expression across libraries was observed.

### Quantitative RT-PCR analysis

To validate our 454 sequencing data, eight genes including hemocyanin (RL-all_rep_c8318), glycine N-methyltransferase (RL-all_rep_c13364), nucleoside diphosphate kinase (RL-all_rep_c11491), lectin (RL-all_rep_c12149), cathepsin L (RL-all_c1576), trypsin (RL-all_c1114), zinc proteinase (RL-all_c2336), and acidic mammalian chitinase (RL-all_rep_c12797) were selected randomly for quantitative RT-PCR analysis, using the same RNA samples as for the 454 sequencing. First strand cDNA was synthesized from 1 µg of RNA using M-MuLV reverse transcriptase (Qiagen). The qPCR reaction mixture (20 µL) consisted of 2× Power SYBR Green PCR Master mix, 0.9 M each of the forward and reverse primers, and 1 µL of template cDNA. PCR amplification was performed under the following conditions: 50°C for 2 min and 95°C for 30 s, followed by 40 cycles of 95°C for 15 s and 62°C for 1 min, and a final extension at 72°C for 5 min. Primer sets were designed using the Primer Premier 5.0 software (Table S1), and the parallel reactions using beta-actin primers [33] were performed to normalize the amount of template cDNA added to each reaction.

## Results and Discussion

### Sequencing and *de novo* assembly

As described in the Methods, cDNA libraries from pooled mRNAs extracted from the hepatopancreas of twenty WSSV-challenged shrimp or of twenty control shrimp were subjected to a 1/2-plate production run on the 454 GS-FLX sequencing instrument, resulting in 159,742 and 101,725 raw reads, respectively. Files containing these data were deposited in the Short Read Archive (SRA) database of the National Center for Biotechnology Information (NCBI) with accession numbers of SRR554363 (WSSV- challenged) and SRR554364 (control). After removal of repetitive, low-complexity, and low-quality reads, a total of 151023 cleaned reads, ranging from 70 bp to 611 bp were harvested in the WSSV-challenged library, with an average length of 374 bp and N50 length of 451 bp. In the control library, a total of 95372 cleaned reads, ranging from 70 bp to 620 bp were obtained, with an average length of 370 bp and N50 length of 444 bp (Table 1). The overall de novo assembly was performed using the combined reads from the two libraries, generating a total of 14538 unigenes. The length distribution of these unigenes is shown in Table 2. The lengths of the unigenes range from 100 to 8750 bp, with the average length of 574 bp, a greater number than that obtained by Illumina sequencing in *L. vannamei* [34]. The relatively long read lengths allow for greater statistical confidence in the *de novo* assembly [35], [36].

**Table 1:** Summary of reads in WSSV-infected and control *L. vannamei* cDNA libraries.

	WSSV-infected	control
Total sequenced cDNA	159742	101725
Cleaned reads	151023	95372
Average length	374 bp	370 bp
N50 length	451 bp	444 bp
Minimum	70 bp	70 bp
Maximum length	611 bp	620 bp

**Table 2:** Length distribution of the unigenes assembled from the combined reads.

Length (bp)	Number of sequences	Percentage
100–500	6672	45.89%
500–1000	6710	46.15%
1000–1500	834	5.74%
1500–2000	218	1.50%
≥2000	104	0.72%
Total	14538	

### Similarity searches

**Figure 1:**
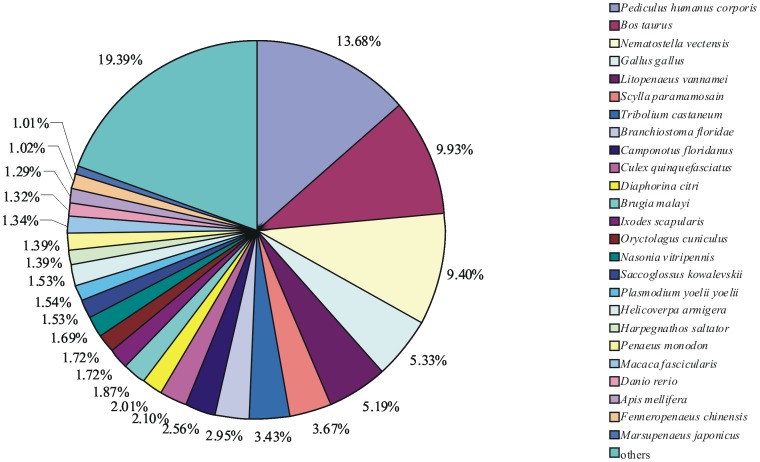
Species distribution of the BLASTX results. The figure shows the species distribution of unigene BLASTX results against the NCBI non-redundant protein databases with a cutoff E value <10^−5^. Different colors represent different species. Species with proportions of more than 1% are shown.

For functional annotation, all unigenes were subjected to BLASTX similarity search against the NCBI non-redundant (NR) protein databases with a cutoff E-value <10^−5^. Among the 14538 unigenes from both the WSSV-infected library and the control library, 4605 (31.68%) showed significant matches in the UniProtKB/Swiss-Prot database. An additional 10625 (73.08%) unigenes showed significant matches in the NR database. In total, 10648 (73.24%) unigenes were annotated in UniProtKB/Swiss-Prot or NR database. The species distribution of the best match result for each sequence is shown in Figure 1. The *L. vannamei* unigenes showed 13.68% matches with *Pediculus humanus corporis* sequences followed by *Bos taurus* (9.93%) and *Nematostella vectensis* (9.40%). Unexpectedly, only 5.19% unigenes were found to be similar to the proteome of *L. vannamei*. This might be attributed to the reason that the amount of the *L. vannamei* protein sequences available in the NR protein databases is relatively limited, and the number of matched sequences does not completely reflect the phylogenetic relationships.

### Transcriptome comparison between WSSV-infected and uninfected shrimp

**Figure 2:**
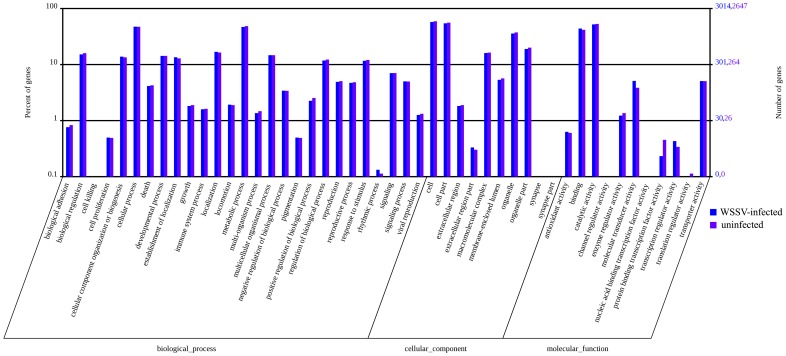
Gene Ontology (GO) classification of putative functions of unigenes from WSSV-infected and uninfected (control) *L.* *vannamei*. The x axis shows subgroups of molecular functions from GO classification and the y axis shows the number of the matched unigenes.

GO is an international standardized gene functional classification system which offers a dynamic-updated controlled vocabulary and a strictly defined concept to comprehensively describe properties of genes and their products in any organis [37]. To obtain an overview of the functional categories represented by the *L. vannamei* transcriptome, we used the GO assignment to classify the functions of the unigenes.Comparison of the transcriptome pattern of the hepatopancreas of WSSV-infected shrimp for GO terms with those of the hepatopancreas of uninfected shrimp showed high similarity in the distribution of unigenes across GO categories. In total, 7325 (45.4%) and 6527 (45.5%) unigenes map to biological processes, 5672 (35.2%) and 5108 (35.6%) unigenes map to cellular components, and 3129 (19.4%) and 2725 (20.0%) unigenes map to molecular functions in the WSSV-infected and uninfected shrimp, respectively (Figure 2). In both infected and uninfected shrimp, most of the corresponding biological process genes were involved in cellular processes, metabolic processes and localization; most of the cellular component genes encode proteins associated with parts of cells and cell organelles; most of the molecular function genes were associated with catalytic activity, binding and molecular transducer activity. The result showed that the functional distribution of the genes of the two shrimp groups was similar, indicating that the number of genes expressed in each GO category was not significantly affected by WSSV-infection.

### Identification of differentially expressed genes

The significant differences in the expression of different unigenes were determined by comparing the relative transcript abundance of each unigene between the WSSV-infected shrimp and uninfected shrimp. We identified 767 host genes that are significantly up-regulated and 729 genes that are significantly down-regulated by infection with WSSV (Table S2). For functional annotation, all the differentially expressed unigenes were searched against NR, Nt, UniProtKB/Swiss-Prot, GO, COG, and KEGG databases by BLASTX with a cut-off E-value of 10^−5^ (Table S2). By this method, 1208 (80.7%) returned an above cut-off BLAST result. Of them, 1206 unigenes were annotated by NR (80.6%), and 819 (54.7%), 840 (56.1%), 534 (35.7%) and 758 (50.7%) unigenes by Nt, UniProtKB/Swiss-Prot, GO and COG, respectively. All differentially expressed unigenes with their Nr, Nt, UniProtKB/Swiss-Prot, COG, GO and ORF analysis are presented in additional Table S2. It is noteworthy that of the 1496 differentially expressed unigenes (up- and down-regulated), 288 (19.3%) unigenes have low sequence homology to known sequences in public databases, suggesting that they may represent non-coding RNA, misassembled contigs or unknown genes of *L. vannamei* involved in response to WSSV-infection.

**Figure 3:**
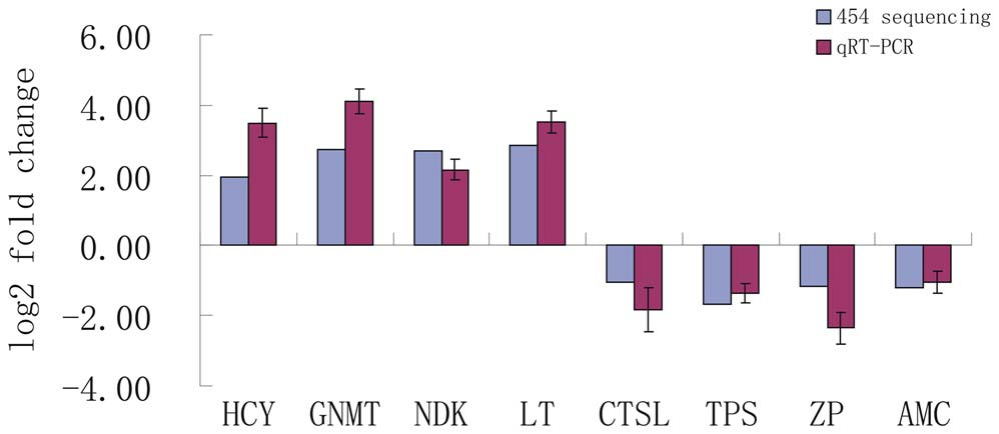
Comparison of the expression profiles of selected genes as determined by 454 sequencing (blue) and qRT-PCR (red). Target gene abbreviations are as follows: HCY - hemocyanin, GNMT - glycine N-methyltransferase, NDK - nucleoside diphosphate kinase, LT - lectin, CTSL - cathepsin L, TPS - trypsin, ZP - zinc proteinase, AMC - acidic mammalian chitinase.

To validate our RNA-seq results, eight unigenes were selected randomly for quantitative real time-PCR (qRT-PCR) analysis. The qRT-PCR results confirmed the data obtained from 454 sequencing analysis showing similar trends in up- or down-regulation of host genes (Figure 3). For example, based on 454 sequencing analysis, hemocyanin, glycine N-methyltransferase, nucleoside diphosphate kinase and lectin were up-regulated 1.94, 2.74, 2.71 and 2.87 log2-fold, respectively, and showed 3.50, 4.12, 2.16 and 3.52 log2-fold changes, respectively in qRT-PCR analyses (Figure 3). Likewise, in 454 sequencing analysis, cathepsin L, trypsin, zinc proteinase and acidic mammalian chitinase were down-regulated −1.04, −1.69, −1.18 and −1.20 log2-fold, respectively, and showed −1.85, −1.35, −2.36 and −1.05 log2-fold changes, respectively in qRT-PCR analyses. Although the results of qRT-PCR do not match the 454 sequencing analysis perfectly, which might be caused by the sequencing biases [6], [23], the qRT-PCR analysis confirmed the change direction detected by the 454 sequencing analysis.

### KEGG analysis of the differentially expressed genes

**Figure 4:**
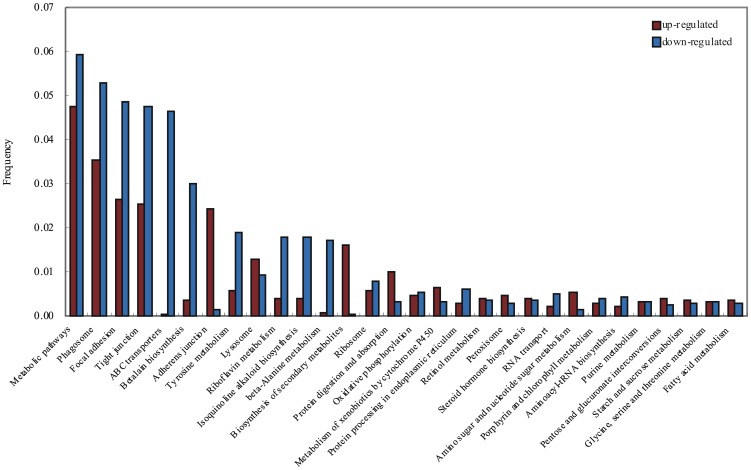
KEGG analysis of up- and down-regulated genes. The top 30 pathways are shown. The x axis shows pathways from KEGG classification and the y axis shows the frequency of the matched unigenes.

KEGG is a pathway-based categorization of orthologous genes that provides useful information for predicting functional profiles of genes [38]. To analyze the interplay between the up- and down-regulated genes, all the differentially expressed genes were mapped to the referential canonical pathways in the Kyoto Encyclopedia of Genes and Genomes (KEGG) database. The top 30 pathways are presented in Figure 4. The distribution of gene pathways between the up- and down-regulated genes is quite different. Genes related to ‘Metabolic pathways’, ‘Phagosome’, ‘Focal adhesion’, ‘Tight junction’, ‘Adherens junction’ and ‘Biosynthesis of secondary metabolites’ were concentrated in the up-regulated gene cohort. Among these, more than 10 fold as many genes related to ‘Adherens junction’ and ‘Biosynthesis of secondary metabolites’ were present in the up-regulated gene group as compared to the group of down-regulated genes. While genes related to ‘Metabolic pathways’, ‘Phagosome’, ‘Focal adhesion’, ‘Tight junction’, ‘ABC transporters’, ‘Betalain biosynthesis’, ‘Tyrosine metabolism’, ‘Riboflavin metabolism’, ‘Isoquinoline alkaloid biosynthesis’, and ‘beta-Alanine metabolism’ were more suppressed in the presence of the viruses. Among these, the number of genes related to ‘ABC transporters’, ‘Betalain biosynthesis’ and ‘beta-Alanine metabolism’ were much larger (9–103 fold) in the down-regulated gene group as compared to the up-regulated genes group. Interestingly, analysis of the distribution of genes by pathways defined by the KEGG revealed clustering of the differentially expressed genes in cell junction pathways. Cell junctions include ‘focal adhesions’, ‘tight junctions’, ‘gap junctions’, ‘adherens junctions’, ‘desmosomes’, and ‘hemidesmosomes’, and each one plays a specific role in adhesion, communication, and transduction of mechanical force. Cell junctions regulate many normal biological processes, such as cell and metabolite transport across epithelial layers, development and differentiation programs, excitable cell contraction, immune responses, and neural activity [39]. Our results suggest that cell junctions might play an important role in the immune responses of shrimp against viral infection.

### Candidate genes involved in *L. vannamei* immune response

Among the genes that were found to be differentially expressed in the WSSV-infected shrimp compared to the uninfected controls, several are involved in various processes of animal defense against pathogens such as apoptosis, MAPK signaling, toll-like receptor, Wnt signaling and antigen processing and presentation (Table 3). Apoptosis pathway is known to be related to the cell hypersensitivity response, blocking pathogen progression and systemic resistance [40], [41]. Apoptosis has been reported to play an essential role in animal defense against viral pathogens. In crustaceans, the occurrence of apoptosis upon viral infection has long been observed [42]. Upon WSSV infection, apoptosis has been detected in several viral target tissues of shrimp [43]. Several studies have shown the changes in the level of apoptosis-related gene expression in shrimp against viral-infection [44]–[47]. In this study, we found six differentially expressed genes, including beclin, catalase, oncoprotein nm23, cathepsin B, deoxyribonuclease I, prohibitin and caspase, are involved in apoptosis. These genes might respond to the WSSV infection through controlling the extent of the apoptosis in the defense response, but the detailed mechanisms still need further investigation. Mitogen-activated protein kinase (MAPK) signaling is another noteworthy pathway. MAPK is an important process for animal defense against pathogens, and was activated during virus infection and contributed to virus replication in animal cells [48]. Among the differentially expressed genes, we found heat shock protein 70, max protein and P38 mapk shared homology to signaling molecules of the MAPK signaling pathway. These genes were over-expressed in the WSSV-infected shrimp, with the fold changes (log2 ratio) ranging from 1.15 to 4.03, indicating they might play an important role in response to WSSV infection. However, the underlying molecular mechanisms also remain unclear. To our knowledge, this is the first report showing the changes in the level of MAPK-related gene expression in WSSV-infected shrimp. Several differentially expressed genes seem to be involved in toll-like receptor (TLR) signaling pathway. TLRs are a class of proteins that have been shown to play a critical role in the activation of innate immunity by recognizing distinct pathogen-associated molecular patterns (PAMPs) derived from viruses, bacteria and fungi [49]. However, there are few reports of interactions between viruses and the TLR signaling pathway in crustacean. Here we found that polyubiquitin-C and caspase, belonging to TLR signaling pathway, were both up-regulated after WSSV infection. Several other genes seem to belong to other immune-relevant pathways, such as Wnt signaling pathway (including ring box protein, transmembrane receptor, and caprin-1) and antigen processing and presentation pathway (including cathepsin L, cathepsin B, autoantigen, legumain, and gamma-interferon-inducible lysosomal thiol reductase). These pathways have also been reported to play critical role in animal defense against viral pathogens [50], [51].

**Table 3:** Candidate genes involved in *L. vannamei* immune response.

Category or gene ID	Homologous function^a^	Species	FC^b^
**Apoptosis pathway**			
RL-all_c360	Beclin	*Pediculus humanus corporis*	1.43
RL-all_c13	Catalase	*Fenneropenaeus chinensis*	−2.08
RL-all_rep_c11491	Oncoprotein nm23	*Litopenaeus vannamei*	2.71
RL-all_rep_c8488	Cathepsin B	*Litopenaeus vannamei*	2.02
RL-all_c667	Deoxyribonuclease I	*Litopenaeus vannamei*	−1.02
RL-all_c6511	Prohibitin	*Litopenaeus vannamei*	−2.02
RL-all_c58	Caspase	*Fenneropenaeus chinensis*	1.46
**MAPK signaling pathway**			
RL-all_rep_c8373	Heat shock protein 70	*Litopenaeus vannamei*	1.15
RL-all_c1444	Max protein	*Apis mellifera*	2.24
RL-all_c5388	P38 mapk	*Aedes aegypti*	4.03
**Toll-like receptor pathway**			
RL-all_c191	Polyubiquitin-C	*Branchiostoma floridae*	1.89
RL-all_c58	Caspase	*Fenneropenaeus chinensis*	1.46
**Wnt signaling pathway**			
RL-all_c1445	Ring box protein	*Bombyx mori*	−1.15
RL-all_c8036	Transmembrane receptor	*Ixodes scapularis*	−1.56
RL-all_c458	Caprin-1	*Harpegnathos saltator*	−1.57
**Antigen processing and presentation pathway**			
RL-all_rep_c11429	Cathepsin L	*Penaeus monodon*	1.02
RL-all_rep_c8488	Cathepsin B	*Litopenaeus vannamei*	2.01
RL-all_c7990	Autoantigen	*Rhipicephalus appendiculatus*	1.02
RL-all_rep_c12441	Legumain	*Haemaphysalis longicornis*	1.18
RL-all_rep_c12517	Gamma-interferon-inducible lysosomal thiol reductase	*Penaeus monodon*	−1.88

a The functions of homologous gene.

b FC, fold changes (log2 ratio) in gene expression.

## Conclusion

Understanding the molecular interactions between *L. vannamei* and WSSV is useful, not only for studies on the molecular responses and defense mechanism in shrimp against pathogens, but also for agricultural practice that aims to develop effective strategies to prevent viral disease. However, these studies have been hampered by the lack of genomic resources of *L. vannamei*. In this study, we investigated the transcriptome profile of WSSV-infected *L. vannamei* using the 454 RNA-seq technology. In conclusion, this study allowed us to (1) Obtain over 14538 unigenes from *L. vannamei*, (2) Compare the transcriptomes between WSSV-infected and uninfected shrimp, and (3) Identify potential pathways and candidate genes involved in *L. vannamei* response to WSSV infection. Further studies on the functionality of these genes will provide valuable information on the antiviral mechanism in shrimp. Furthermore, the large number of transcripts obtained in this study provides a strong basis for future genomic research on shrimp.

## Supporting Information

Table S1 **Primers used in qRT-PCR for validation of differentially expressed genes.**
(XLS)

Table S2 **List of differentially expressed genes with Nr, Nt, UniProtKB/Swiss-Prot, GO, COG and ORF analysis.**
(XLS)
